# Primary health care nurses’ knowledge, self-efficacy and performance of diabetes self-management support

**DOI:** 10.4102/phcfm.v15i1.3713

**Published:** 2023-01-25

**Authors:** Zandile K. Landu, Talitha Crowley

**Affiliations:** 1 Department of Nursing and Midwifery, Faculty of Medicine and Health Sciences, Stellenbosch University, Cape Town, South Africa; 2 School of Nursing, Faculty of Community and Health Sciences, University of the Western Cape, Cape Town, South Africa

**Keywords:** Diabetes mellitus, knowledge, nurses, diabetes self-management support, self-efficacy, self-management

## Abstract

**Background:**

Patients living with diabetes are primarily managed and supported by nurses in primary health care (PHC). Therefore, PHC nurses require knowledge of diabetes and confidence (self-efficacy) to perform diabetes self-management support (SMS).

**Aim:**

This study evaluated the diabetes knowledge, self-efficacy and performance of diabetes SMS by PHC nurses.

**Setting:**

Primary health care facilities in King Sabata Dalindyebo subdistrict, O.R. Tambo district, Eastern Cape.

**Methods:**

A quantitative cross-sectional and simple correlational design was used. Registered nurses (*n* = 100) completed a validated self-reporting questionnaire to measure diabetes knowledge, self-efficacy and performance of SMS.

**Results:**

Participants’ diabetes knowledge mean scores were high (mean of 11.9, standard deviation [s.d.] 1.8, out of 14). Self-efficacy scores (mean 18.91, s.d. 3.2 out of 24) were higher than performance of SMS scores (mean 17.81, s.d. 3.3 out of 24). Knowledge was not associated with self-efficacy or performance, but self-efficacy was positively correlated with performance of SMS (*r* = 0.78, *p* < 0.01). Nurses with a postgraduate qualification in primary care nursing had significantly higher diabetes knowledge scores (mean = 92.9 vs. 83.8; *p* = 0.03), and years of experience as a nurse were positively correlated with the performance of SMS (*r* = 0.21, *p* = 0.05).

**Conclusion:**

Diabetes knowledge of PHC nurses in this study does not translate into self-efficacy and the performance of SMS in practice, indicating the need for specific SMS training, support by experienced mentors, appropriate guidelines and comprehensive integrated chronic care systems.

**Contribution:**

This is the first study to report on the SMS self-efficacy and performance of PHC nurses in South Africa.

## Introduction

The increasing mortality rate of people living with diabetes is a global concern. According to the International Diabetes Federation, South Africa ranks amongst the top five countries with the highest diabetes incidence in Africa, with an age-adjusted (20–79 years) comparative incidence of 10.8%.^1^ Diabetes mellitus is a metabolic condition characterised by elevated blood sugar levels (hyperglycaemia) and metabolic disturbances of carbohydrates, fat and protein, which result from defects in insulin secretion, action or both.^2^

The rise in noncommunicable chronic diseases (NCDs) such as diabetes compelled countries like South Africa to introduce an integrated chronic disease model (ICDM) for primary health care (PHC).^3^ According to this model, 70% – 80% of persons living with chronic diseases can be managed in communities through ‘assisted self-management support and population level awareness’.^3^ Assisted self-management support (SMS) focuses on health promotion and education at the community level, identification of patients at risk at the household level, point of care testing and screening, support groups and medication delivery.^3^

Self-management is an ongoing process where individuals are actively involved on a daily basis in the management of their chronic condition.^4^ For patients to self-manage, nurses must provide SMS, in which they educate patients, giving them skills with the aim of conserving or enhancing health and a patient’s self-efficacy towards goal achievement.^5^ Self-management support is one of the four components of the chronic care model (CCM) of Wagner that provides a framework for improving the quality of chronic care and directs the implementation of chronic healthcare services.^6^ Self-management support has been proven to improve patient outcomes such as Hemoglobin A1c (HbA1C).^5,7,8^ The ‘5 A’s’ approach of assess, advise, agree, assist and arrange is used to guide the implementation of SMS in patients with a chronic condition.^9^

Studies conducted in sub-Saharan Africa show that self-management is generally poor and a serious threat to the health of individuals living with diabetes.^10^ There are various reasons for this. In Uganda, for example, SMS is not prioritised because of a shortage of healthcare workers.^10^ In South Africa, the main challenges to self-management faced by people living with diabetes were related to financial constraints, prolonged waiting times in health centres and the limited care and management skills of healthcare workers.^11^ Lack of trained personnel, accurate guidelines and commitment to SMS were all found to be factors that affected the quality of care given to people living with diabetes in a study conducted in Sweden, South Africa and Uganda.^12^

Currently, there is no structured self-management programme in South Africa. Although the ICDM has a component called ‘assisted self-management’, which is primarily provided by ward-based PHC outreach teams, there are no specific guidelines for nurses or other healthcare workers on how to provide SMS.^13^ To provide diabetes SMS and oversee community health workers (CHWs), professional nurses in PHC need knowledge of diabetes and its management as well as teaching and counselling skills.^14^ However, nurses are seldom trained to provide SMS.^14^ An investigation into PHC nurses’ knowledge, self-efficacy and performance of diabetes SMS may highlight the gaps to inform training, which is critical for the success of the ICDM. The study aim was to evaluate diabetes knowledge, self-efficacy and performance of diabetes SMS by PHC nurses. Diabetes knowledge was positively hypothesised to be associated with self-efficacy and performance of diabetes SMS.

## Research methods and design

### Study design and setting

A quantitative cross-sectional and simple correlational design was used to investigate the associations between nurses’ diabetes knowledge and SMS self-efficacy and performance of SMS. The study was conducted in PHC clinics and community health centres (CHCs) in King Sabata Dalindyebo (KSD) subdistrict in the O.R. Tambo District, Eastern Cape. King Sabata Dalindyebo was selected as it is the largest subdistrict in O.R. Tambo District with 49 clinics and five CHCs.

### Study population and sampling strategy

Professional nurses rendering PHC services in 17 KSD clinics were the study’s target population (*N* = 167). Because of the relatively small population, exhaustive sampling was used. Operational managers and community service nurses were excluded because of their limited experience in rendering clinical care. Nurses working in coronavirus disease 2019 (COVID-19) vaccination roving teams could not be accessed at the time. Therefore, out of 167 nurses, 143 potential participants who were available at the time of the study were approached, of whom only 125 met the inclusion criteria for the study and 106 consented to participate. Out of the 106, six questionnaires were discarded, leaving a final sample of 100. This sample size was deemed sufficient, as a sample size of 100 from a population of 167 produces a two-sided 95% confidence interval with a precision (half-width) of 0.06 when the actual proportion is near 0.5.

### Data collection and instrumentation

The first author and a fieldworker collected data between July and August 2021. Since data were collected in PHC facilities, COVID-19 regulations were followed.

The self-administered questionnaire comprised three sections: (1) demographic questions; (2) the Diabetes Basic Knowledge Test (DBKT) adapted from the modified version of the ‘Diabetes Knowledge Test’,^15^ and (3) the Self-efficacy and Performance in Self-Management Support (SEPSS) instrument.^16^

The original DBKT contained 52 multiple-choice questions coded as 0 = incorrect, 1 = I don’t know and 2 = correct. In order to reduce the questionnaire length and contextualise the instrument, the items were reduced to 14 items that focused on type 1 and 2 diabetes mellitus aetiology, management of the disease and effects of insulin, including physiological action and storage. Two local experts reviewed the adapted instrument to assess relevancy, alignment with local guidelines and contextual appropriateness. A knowledge score was created by adding the number of correct responses (total out of 14).

The SEPSS is a validated instrument containing 36 items in six subscales (Assess, Advise, Agree, Assist, Arrange and Partnership) that measures SMS self-efficacy and performance separately on a four-point Likert scale. Scoring of the SEPSS instrument required that participants rate both self-efficacy and performance on the same set of items. Scores range from 0 to 4 for the subscales and from 0 to 24 at a total scale. Higher scores on the SEPSS reflect a higher level of self-efficacy and performance of SMS.

The questionnaire was pilot tested with 16 PHC nurses who attended a diabetes management workshop to evaluate the readability and clarity of the questions and test the data collection procedures. Data were not included in the main study.

Table 1 indicates the previously reported reliability statistics of the instruments and the values in the present study, showing acceptable reliability for the SEPSS self-efficacy and performance scales but low reliability for the DBKT instrument.^15,16^ Normally, Cronbach’s alpha is not used to measure internal consistency of knowledge questions, as participants may be knowledgeable in some areas and lack knowledge in other areas.

**TABLE 1:** Reliability of the data collection instruments.

Instrument	Cronbach’s alpha	ICC (test–retest)	Present study Cronbach’s alpha
DBKT	0.75	-	0.39
SEPSS – Self-efficacy	0.96	0.95	0.89
SEPSS – Performance of SMS	0.95	0.94	0.93

ICC, intraclass correlation coefficient; DBKT, Diabetes Basic Knowledge Test; SEPSS, Self-efficacy and Performance in Self-Management Support; SMS, self-management support.

### Data analysis

Collected data were entered in the Statistical Package for the Social Sciences (SPSS) version 27 (IBM Corporation, Armonk, New York, United States) and analysed. For descriptive statistics, data were summarised; continuous variables such as age, knowledge and self-efficacy scores were reported by the mean, mode and median, depending on whether they were normally distributed or not. For data that were normally distributed, the mean and standard deviation (s.d.) were reported. Frequencies and percentages were calculated based on the number of valid responses (with missing values excluded).

To test for associations between the dependent continuous variables and independent variables with two categories, the Mann–Whitney *U* test was used as the dependent variables were not normally distributed. The Kruskal–Wallis test was used to test for associations between three or more categories. To identify relationships between continuous variables, Spearman’s *r* correlation test was used.

### Ethical considerations

Ethical approval to conduct the study was received from the Health Research Ethics Committee (HREC) at Stellenbosch University (reference number S20/12/349), the Eastern Cape Department of Health (reference number EC_20204_003) and the O.R. Tambo District Health Department. Participants were given a choice of whether to participate or not, and they were informed that they were allowed to withdraw from the study at any point. All participants signed written consent forms. Data were collected anonymously and are being kept in a password-protected folder for five years.

The youngest participant was 24 and the eldest 61 years old (mean 42.3; s.d. 10.7) (not shown on table).

## Results

### Demographics

The demographic data of the participants measured on a nominal or ordinal level are displayed in Table 2. The majority of the participants were female (86%), held a diploma in nursing (59.2%) and had recent experience working with patients living with diabetes (55.1%).

**TABLE 2:** Demographic data.

Variable	Frequency	%
**Gender (*n* = 100)**		
Female	86	86.0
Male	13	13.0
Nonbinary	1	1.0
**Job title (*n* = 99)**		
Professional nurse	94	94.9
Senior professional nurse	5	5.1
**Highest qualification (*n* = 98)**		
Diploma in Nursing	58	59.2
B Cur	24	24.5
Postgraduate	16	16.3
**Postgraduate diploma in PHC (*n* = 98)**		
Yes	10	10.2
No	88	89.8
**Last time worked with patients living with diabetes (*n* = 98)**		
Less than 1 month ago	54	55.1
Less than 3 months ago	11	11.2
3–6 months ago	5	5.1
More than 6 months ago	28	28.6
**Years of working experience as a professional nurse (*n* = 92)**		
1–10	57	62.0
11–20	26	28.2
21–30	9	9.8

PHC, primary health care; B Cur, Baccalaureus Curationis (Bachelor’s Degree).

The youngest participant was 24 and the eldest 61 years old (mean 42.3; s.d. 10.7) (not shown on table).

### Diabetes knowledge

The results of the knowledge questions are displayed in Table 3. Forty-five participants (45%) did not answer the question regarding causes of hyperglycaemia. Other questions with a frequency of correct responses below 80% were: needle contamination (77%), physiological action of insulin (78%) and insulin storage (79%). The minimum knowledge score was 7 and the maximum 14. The mean score was 11.9 (s.d. 1.8). All the participants had a score of ≥ 50%. Out of 100 participants, 75 had a knowledge of more or equal to 75%. Only 64 of the participants had a knowledge score of more than or equal to 80%.

**TABLE 3:** Frequency of correct knowledge responses.

Variable	Correct response frequency	%
Aetiology	82	82.0
Tests to monitor diabetes control	87	87.0
Signs of hyperglycaemia	83	83.0
Cause of hyperglycaemia	45	45.0
When do you check for ketones?	83	83.0
Signs of hypoglycaemia	83	83.0
Cause of hypoglycaemia	90	90.0
Insulin storage	79	79.0
Normal fasting blood glucose level	95	95.0
What guides your initial actions if a diabetic person is found unresponsive?	96	96.0
Physiological actions of insulin	78	78.0
Action to take if needle is contaminated	77	77.0
What effect does insulin have on blood glucose?	89	89.0
The most appropriate statements about management of type 2 diabetes	82	82.0

### Self-efficacy and performance in self-management support

The mean item responses to the items in the SEPSS instrument are indicated in Table 4.

**TABLE 4:** Self-efficacy and performance of self-management mean scores.

Variable		Self-efficacy		Performance	
	*n*	Mean	s.d.	Mean	s.d.
**Subscale: Assess**					
Ask the patient what he or she thinks about living with diabetes in the future	100	3.0	1.0	3.2	0.8
Ask what patient knows	100	3.3	0.8	2.3	1.2
Ask a patient to share his emotions about diabetes	100	2.7	1.1	2.7	1.1
Ask about available motivation and discipline to integrate diabetes in his life	100	2.9	1.0	2.8	1.1
Ask how much confidence he has in his own abilities	100	3.0	1.0	3.2	1.0
Ask what he can and wants to do for himself in his daily care related to diabetes	100	3.4	0.7	3.2	0.9
Total		3.06	0.7	2.81	0.8
**Subscale: Advise**					
Ask patient what information he needs	100	3.2	0.8	3.1	0.9
Ask the patient for permission to give information or advice	100	3.2	0.9	3.0	0.9
Letting the patient restate information given	100	3.2	0.8	3.0	0.9
Giving the patient education and instruction about the chronic condition	100	3.4	0.8	3.6	0.7
Helping the patient to formulate questions to discuss with healthcare workers	100	2.9	1.1	2.6	1.2
Involving the family when giving information and winstruction	100	3.0	1.1	2.6	1.2
Total		3.16	0.6	2.98	0.6
**Subscale: Agree**					
Search for earlier positive experiences in achieving goals	100	2.7	1.3	2.4	1.3
Let the patient prioritise when setting goals	100	2.9	0.9	2.8	1.1
Developing a plan of action to achieve goals with patient	100	3.0	1.0	2.9	1.0
Document the goals and agreements in patient record	100	3.1	1.1	3.0	1.3
Help patient to make decisions concerning treatment	100	3.3	1.0	3.3	0.8
Recognise patient uncertainty about making a treatment decision	100	3.3	0.8	3.2	0.9
Total		3.05	0.7	2.93	0.7
**Subscale: Assist**					
Discuss with patient who he will inform about his chronic condition	100	3.5	0.8	3.4	0.9
Encourage the patient to perform as many daily activities as possible	100	3.5	0.6	3.5	0.7
Helping the patient to choose the activities that he can realistically do	100	3.2	0.8	3.1	0.9
Discuss with the patient who can provide daily support	100	3.2	1.0	3.0	1.0
Discuss with patient how he can make use of self-management assistive devices daily	100	2.9	1.1	2.7	1.1
Assist patient to monitor his own health and physical reactions	100	3.3	0.9	3.2	0.9
Total		3.33	0.6	3.18	0.6
**Subscale: Arrange**					
Ask about convenient time for follow-up care	100	3.4	0.8	3.2	1.5
Inform and coordinate with other health care professionals	100	3.2	0.8	3.2	0.9
Using assistive devices and technology to provide remote guidance to the patient	100	2.7	1.1	2.3	1.3
Facilitating the patient to easily stay in contact between appointments	100	3.5	0.8	3.2	1.1
Initiating contact between appointments to discuss health and solve difficulties	100	3.0	1.1	2.8	1.1
Examining progress of care plan actions together with the patient	100	3.3	0.9	3.1	0.9
Total		3.20	0.6	2.99	0.8
**Subscale: Partnership**					
Accepting patient experience as valuable information concerning care delivery	100	3.0	0.9	3.0	1.1
Consider cultural background of the patient	100	3.2	1.0	2.7	1.0
Determine together with patient how much of the care coordination I take for him	100	3.0	0.9	2.4	1.3
Using the patient choices as the basis for care, even if it’s not ideal from a medical view	100	2.6	1.3	3.1	1.1
Showing empathy when patient does not succeed in achieving the established goals	100	3.2	1.0	3.2	0.9
Reflecting upon my own practice	100	3.4	0.8	3.0	1.1
Total		3.11	0.7	2.9	0.7

s.d. Standard deviation.

The subscale with the highest mean for self-efficacy and performance was ‘Assist’ with mean scores of 3.33 and 3.18, respectively.

The subscale with the lowest mean for self-efficacy was ‘Agree’ (mean 3.05), and for performance, it was ‘Assess’ (mean 2.93). Participants had lower self-efficacy in exploring previous positive experiences in achieving goals (mean 2.7) and were the least likely to ask what a person knows during the assessment (mean 2.3).

Participants further had low mean scores for both self-efficacy (mean 2.7) and performance (mean 2.3) for the item related to using assistive devices and technology to provide remote guidance to the patient.

The self-efficacy scale had a higher total mean (18.91, s.d. 3.2) than the performance scale (17.8, s.d. 3.3).

### Associations between knowledge, self-efficacy and performance in self-management support and demographic variables

Participants’ diabetes knowledge mean scores were high, but there was no association between the knowledge score, the total self-efficacy and performance scores (*p* > 0.05). However, there was a strong positive correlation between the total self-efficacy and total performance (*r* = 0.78, *p* < 0.01) (Table 5).

**TABLE 5:** Correlations between knowledge, self-management support self-efficacy and performance.

Spearman’s rho	Knowledge score % performance	Total self-efficacy	Total
**Knowledge score %**			
Correlation coefficient	1.00	−0.03	−0.01
Sig. (2-tailed)	-	0.07	0.03
***N***	100	100	100
**Total self-efficacy**			
Correlation coefficient	0.03	1.00	0.78†
Sig. (2-tailed)	0.75	-	0.00
***N***	100	100	100
**Total performance**			
Correlation coefficient	−0.01	0.78†	1.00
Sig. (2-tailed)	0.32	0.00	-
***N***	100	100	100

† , Correlation is significant at the 0.01 level (2-tailed).

Nurses with a PHC qualification had a significantly higher mean diabetes knowledge score (mean 92.9, s.d. 7.5) compared with those who did not have a PHC qualification (mean 83.8, s.d. 12.9) (Mann–Whitney *U, p* = 0.03). There was a significant positive correlation between the years of experience as a professional nurse and the performance of SMS score (*r* = 0.21, *p* = 0.045). No other significant associations were found between demographic variables, including experience in working with patients living with diabetes and knowledge, self-efficacy and performance of SMS.

## Discussion

Primary health care nurses in this study had high diabetes knowledge scores, with a mean score of 11.9 out of 14 (85%), although only 64 participants had a knowledge score of ≥ 80%. The mean score is much higher compared with the mean score (54.5%) in a study conducted in Southern Carolina, which used the original DBKT tool.^15^ This may be because we used an abbreviated version of the DBTK tool. In addition, we only included questions considered essential for managing patients in PHC settings and therefore expected high knowledge scores. An integrative review on nurses’ diabetes knowledge that included studies from Africa, Australia, the United States of America and Europe, found that nurses had insufficient understanding of diabetes pathophysiology, symptoms and management, with markedly different results between studies.^17^ Conversely, 96% of participants in the present study knew what action to take if a patient is unconscious, 83% understood the signs of hypoglycaemia, 90% knew the causes of hypoglycaemia and 78% were aware of the physiological action of insulin. The question with the lowest score (45%) was the causes of hyperglycaemia. Knowledge of the causes of hyperglycaemia is crucial to patient education and management of medical emergencies in patients living with diabetes. This knowledge deficit represents a major risk for unsafe practice.^1,17^ Adequate knowledge is important as it is thought to influence self-efficacy and performance of self-management, thereby improving patient clinical outcomes.^18^

Self-efficacy scores in this study were high (mean 18.91, s.d. 3.1) compared with a study conducted by Duprez et al. to validate the SEPSS tool in the Netherlands (mean 17.2, s.d. = 3.31).^16^ Findings in the study conducted in the Netherlands showed that nurses working in outpatient departments had higher levels of self-efficacy because they work more regularly with patients living with diabetes compared with nurses working in inpatient departments (mean of 18.71 vs. 16.75).^16^ High self-efficacy scores in our study support this finding, as most participants had recent experience in working with patients living with diabetes and are working in PHC, a setting similar to the outpatient department; as such, their self-efficacy scores were high. An Australian study found that low levels of self-efficacy was related to diabetes knowledge deficiencies;^14^ however, in our study we did not find an association between diabetes knowledge and self-efficacy.

Performance of SMS in this study was also higher (17.81, s.d. 3.3) compared with the study conducted by Duprez et al.^16^ (11.75, s.d. 3.8). The ‘assess’ subscale had the lowest score of 2.81 (s.d. 0.8) on the performance scale. This is concerning because patients must be thoroughly assessed in order to plan care that will meet individual patient needs and provide support. Patients with a chronic condition often lack support from healthcare workers to improve coping skills, emotional management and role management.^19^

With the high burden of disease, the use of technology in diabetes self-management has become increasingly important.^20,21^ In our study, participants had low mean scores in the use of assistive devices and technology for SMS. This means that nurses may need training and support on the use of assistive devices and technology for SMS.

The total mean performance in SMS score was slightly lower than the total self-efficacy score (17.81 vs. 18.91). Several factors may inhibit nurses from providing SMS, which include institutional factors and personal factors.^22^ In busy environments with high workloads such as PHC settings, nurses may not prioritise diabetes SMS.^17^ In South Africa, possible factors may include unavailability of guidelines and the lack of structures within the institution that encourage SMS. The guideline mostly used is the South African primary care setting is the adult primary care (APC or primary adult care [PAC]) guide.^23^ The APC or PAC divides the provision of routine chronic care into three phases: Assess, Advise and Treat. We argue a guideline that incorporates all five phases of the five A’s approach, including partnership,^16^ is more suited for person-centred chronic care and may make nurses more aware of the SMS they need to provide.

Inequities between public services and private services remain a challenge in South Africa.^24^ Primary health care services in South Africa do not have enough resources to support nurses to deliver quality care, particularly SMS that is integrated into PHC. As mentioned earlier, there is no formal structured SMS programme in South Africa. The current ‘assistive self-management’ programme only comprises one aspect of SMS, namely medication delivery through Central Chronic Medication Dispensation and Distribution (CCMDD). Although patient support groups through chronic care clubs are part of the ICDM, it is not rolled out in all settings, and there are no guidelines to support healthcare workers to provide SMS in these contexts. An audit of diabetes SMS programmes in South Africa found that patients are educated in the waiting area or during a consultation. This method of delivering SMS is clouded by many challenges, which include time constraints on the provider side, lack of privacy and noise levels.^25^ Further, a systematic review on the integration of SMS into usual care found SMS has the most impact in PHC settings, that SMS should be provided throughout the care continuum and that integration of SMS into care can be classified by the level of engagement of the healthcare provider, for example, high (actively providing SMS), medium (sometimes promoting SMS) and low (aware that SMS is provided elsewhere). Patients who perceived a high level of engagement of their provider had better health-related outcomes.^26^ Therefore, a comprehensive integrated chronic care system with a high level of engagement from nurses is needed.^17^

Patients living with diabetes in PHC settings are primarily seen and managed by nurses. Community-based, nurse-led SMS interventions can improve the health-related outcomes of persons with diabetes if nurses are specifically trained.^5^ Therefore, nurses need to be skilled to manage patients with chronic diseases. In this study, only 10.2% of nurses had a postgraduate diploma in PHC. Nurses with this qualification are more specialised to care for patients living with diabetes than nurses without the qualification and can prescribe treatment according to guidelines.^27^ Although our study did not find an association between diabetes knowledge and self-efficacy and performance of SMS, it may be because nurses lack knowledge and skills in the provision of SMS more specifically. Lack of skilled nurses may affect quality of care and implementation of SMS. Disease self-management needs support from nurses who are well-informed about diabetes and are up to date with the latest evidence-based practices related to diabetes and skills related to diabetes SMS.^15^ Participants in this study with a postgraduate qualification in PHC had higher knowledge scores. This may be because of the intensive training in health assessment, diagnosis, treatment and care, including pharmacology and nondrug treatment (e.g. diet, exercise). In Thailand, nurses with a higher level of education were more confident in managing chronic diseases like diabetes compared with nurses who were less qualified.^28^ However, findings from an integrative review on nurses’ diabetes knowledge found that university education does not guarantee a high level of knowledge and that continuous professional education is needed.^17^ Other factors influencing knowledge include exposure to patients with diabetes and access to knowledgeable practitioners,^17^ although we did not find associations between diabetes knowledge and experience.

We hypothesised that nurses’ diabetes knowledge would be positively associated with self-efficacy and the performance of diabetes SMS. Although knowledge was not associated with SMS self-efficacy or performance, SMS self-efficacy had a strong positive correlation with SMS performance. This means that nurses with self-efficacy may be more likely to perform SMS. The correlation found in the current study (*r* = 0.78) was stronger than the moderate correlation (*r* = 0.63) found by Duprez et al.^16^ The authors indicated that the responses to the two scales differed markedly. The difference in the present study was therefore not as marked.

Years of experience as a professional nurse was correlated with SMS performance, although the correlation was weak (*r* = 0.21). Similarly, in the Netherlands, professional nurses had higher self-efficacy and performance scores than nursing students.^16^ Mentoring for SMS by skilled and experienced professionals might therefore be another strategy to improve the implementation of SMS.

### Strengths and limitations

To the best of our knowledge, this is the first study to assess diabetes SMS self-efficacy and performance in South Africa using previously validated tools. Limitations include convenience sampling and that some nurses were not available because of the COVID-19 pandemic and vaccination campaigns. To compensate, several attempts were made to include all available participants. The DBKT tool had questionable reliability even though it was validated by local experts and pilot tested. This may be because the participants did not have consistent levels of knowledge across the diabetes knowledge items and the number of items was reduced to shorten the questionnaire. The inherent limitation of self-report measures is subjectivity, and it may not reflect actual practice. This, however, further emphasises the need to educate nurses regarding the provision of SMS. The difference between the self-efficacy and performance of SMS scores reported in this study may not be of clinical significance. Data may not be generalisable outside the O.R. Tambo District.

## Conclusion

Primary health care nurses in O.R. Tambo have high levels of diabetes knowledge; however, this does not translate into SMS self-efficacy and performance. Nurses need support to implement SMS through appropriate guidelines, education, training and mentoring, as well as comprehensive integrated chronic care systems.
